# Recovery of healthy sexuality in patients with Anorexia Nervosa treated with Enhanced Cognitive Behaviour Therapy (CBT-E): results from a two-year follow-up study highlighting the role of avoidant attachment style

**DOI:** 10.1192/j.eurpsy.2022.224

**Published:** 2022-09-01

**Authors:** E. Cassioli, E. Rossi, C. Vizzotto, V. Malinconi, L. Vignozzi, V. Ricca, G. Castellini

**Affiliations:** 1 University of Florence, Psychiatry Unit, Department Of Health Sciences, Florence, Italy; 2 University of Florence, Sexual Medicine And Andrology Unit, Department Of Experimental, Clinical, And Biomedical Sciences “mario Serio”, Florence, Italy

**Keywords:** Cognitive Behaviour Therapy, Anorexia nervosa, female sexuality, adult attachment style

## Abstract

**Introduction:**

There is a known association between the core psychopathological features of anorexia nervosa (AN) and sexual dysfunctions, to the point that the recovery of healthy sexuality could be considered a marker of recovery. However, no studies have evaluated the role of insecure attachment in moderating this recovery during treatment.

**Objectives:**

To evaluate the role of insecure attachment as a possible moderator of the recovery of healthy sexuality in patients with AN treated with Enhanced Cognitive Behaviour Therapy (CBT-E).

**Methods:**

A total of 65 patients with anorexia nervosa were treated with CBT-E in a multidisciplinary environment, after filling out self-administered questionnaires for the evaluation of general (SCL-90-R) and ED-specific psychopathology (EDE-Q), female sexuality (FSFI) and adult attachment style (ECR). The assessment was repeated after one (T1) and two years (T2).

**Results:**

At baseline, all domains of sexual dysfunction were significantly predicted by avoidant attachment. A significant amelioration of both general and eating disorder-specific psychopathology and sexual dysfunctions was observed at all follow-up evaluations with respect to baseline levels. However, only 45% of remitted patients also showed a complete recovery of healthy sexuality: this subgroup reported significantly lower avoidance scores when compared to patients who only recovered from AN. Moderation analysis indicated that sexual desire did not increase in participants with higher levels of avoidant attachment.

**Figure:**
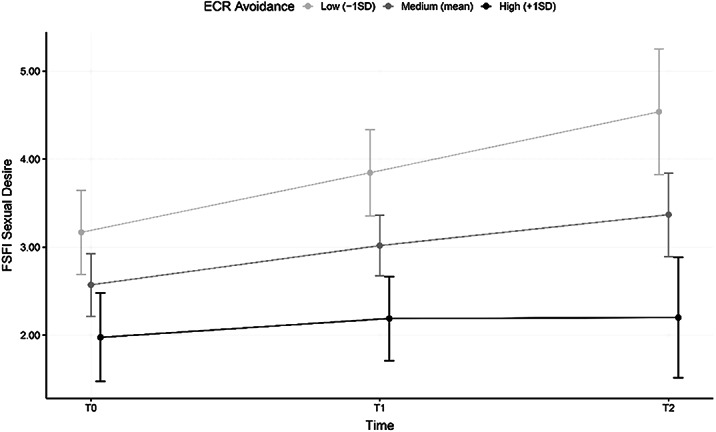

**Conclusions:**

This study highlighted the crucial role of avoidant attachment in the relationship between AN and sexual dysfunctions, underlining the importance of assessing adult attachment for a better characterization and treatment. Attachment-focused interventions may be beneficial for a full recovery.

**Disclosure:**

No significant relationships.

